# Uncovering sperm metabolome to discover biomarkers for bull fertility

**DOI:** 10.1186/s12864-019-6074-6

**Published:** 2019-09-18

**Authors:** E. B. Menezes, A. L. C. Velho, F. Santos, T. Dinh, A. Kaya, E. Topper, A. A. Moura, E. Memili

**Affiliations:** 1 Department of Animal and Dairy Sciences, Mississippi State University, 4025 Wise Center, Mississippi State, MS 39762 USA; 2 Department of Animal Sciences, Federal University of Ceara, Fortaleza, Brazil; 3 Department of Reproduction and Artificial Insemination, Selcuk University, Konya, Turkey; 4 Alta Genetic Inc., Watertown, WI USA

**Keywords:** Metabolomics, Bovine, Spermatozoa, Gas chromatography, Mass spectrometry

## Abstract

**Background:**

Subfertility decreases the efficiency of the cattle industry because artificial insemination employs spermatozoa from a single bull to inseminate thousands of cows. Variation in bull fertility has been demonstrated even among those animals exhibiting normal sperm numbers, motility, and morphology. Despite advances in research, molecular and cellular mechanisms underlying the causes of low fertility in some bulls have not been fully elucidated. In this study, we investigated the metabolic profile of bull spermatozoa using non-targeted metabolomics. Statistical analysis and bioinformatic tools were employed to evaluate the metabolic profiles high and low fertility groups. Metabolic pathways associated with the sperm metabolome were also reported.

**Results:**

A total of 22 distinct metabolites were detected in spermatozoa from bulls with high fertility (HF) or low fertility (LF) phenotype. The major metabolite classes of bovine sperm were organic acids/derivatives and fatty acids/conjugates. We demonstrated that the abundance ratios of five sperm metabolites were statistically different between HF and LF groups including gamma-aminobutyric acid (GABA), carbamate, benzoic acid, lactic acid, and palmitic acid. Metabolites with different abundances in HF and LF bulls had also VIP scores of greater than 1.5 and AUC- ROC curves of more than 80%. In addition, four metabolic pathways associated with differential metabolites namely alanine, aspartate and glutamate metabolism, β-alanine metabolism, glycolysis or gluconeogenesis, and pyruvate metabolism were also explored.

**Conclusions:**

This is the first study aimed at ascertaining the metabolome of spermatozoa from bulls with different fertility phenotype using gas chromatography-mass spectrometry. We identified five metabolites in the two groups of sires and such molecules can be used, in the future, as key indicators of bull fertility.

## Background

Fertility is the key to success for sustainability and economics of the livestock system in both beef and dairy cattle industries [1]. In cattle breeding, artificial insemination (AI) is the most common assisted reproductive technology, and it employs ejaculates from genetically superior sires to inseminate a large number of cows [2]. However, only around 50% of such inseminations result in successful pregnancies, leading to considerable economic losses [3, 4]. Some studies reported that a significant percentage of reproductive failure (i.e., low numbers of pregnant females) is caused by the low fertilizing capacity of the male gamete [5, 6]. The accurate evaluation of bull fertility is currently attempted through routine analysis of semen, but such conventional analysis is unable to determine a priori the full potential and actual fertility of the sires [4, 7, 8]. In addition, conventional analysis of semen has limited use for identification and prediction of sub-fertile animals [9]. The molecular events linked to sperm physiology are important because they serve as the foundation for identification of indicators of the fertilizing capacity of sires, improving the outcomes of AI [10].

The complex nature of events involved in fertilization is affected by the fluctuating concentrations of macromolecules found in the spermatozoon itself [11, 12], seminal plasma [13], and by the microenvironment of female reproductive tract [14]. At ejaculation, spermatozoa become coated with seminal plasma proteins, and even though the seminal fluid is diluted into the female reproductive tract, the effects of those molecules are likely to be maintained as they quickly adhere to the sperm surface. Several studies have reported that seminal plasma proteins improve the fertilizing capacity of sperm [15–19]. Furthermore, recent reports provide evidence that the expression of microRNAs in bull spermatozoa is associated with fertility outcomes [20, 21], and proteomics approaches have also been used for the discovery of potential fertility biomarkers. Metabolomics is vitally important because low molecular weight compounds may provide a clear picture of the regulatory pathways within spermatozoa [22, 23]. Also, metabolites are linked to physiological events through a cascade of biochemical complex networks [24, 25] and also contribute to the definition of the phenotype of an individual [25]. Growing evidence suggests that spermatozoa metabolize a wide spectrum of exogenous substrates that directly or indirectly regulate the signaling pathways involved in sperm motility, hyperactivation, capacitation, acrosome reaction, and sperm-oocyte fusion [26].

In recent years, studies using approaches in metabolomics have revealed potential fertility biomarkers in the seminal plasma of humans [27, 28], stallions [12], and bulls [29]. In a recent publication, we reported the identification of 63 compounds in bull seminal plasma by using gas chromatography-mass spectroscopy (GC-MS). Fructose was the most abundant metabolites in the bovine seminal fluid, followed by citric acid, lactic acid, urea, and phosphoric acid [30]. A wide range of metabolites has also been detected in spermatozoa from humans [31–33], bulls [34], boars [35] and goats [36], bringing evidence that both seminal plasma and sperm metabolites could be meaningfully related to the fertility of males. The present study was conducted to test the hypothesis that metabolites of ejaculated sperm were associated with fertility scores of dairy bulls.

## Results

### Metabolome profile of bull spermatozoa

**Fig. 1:**
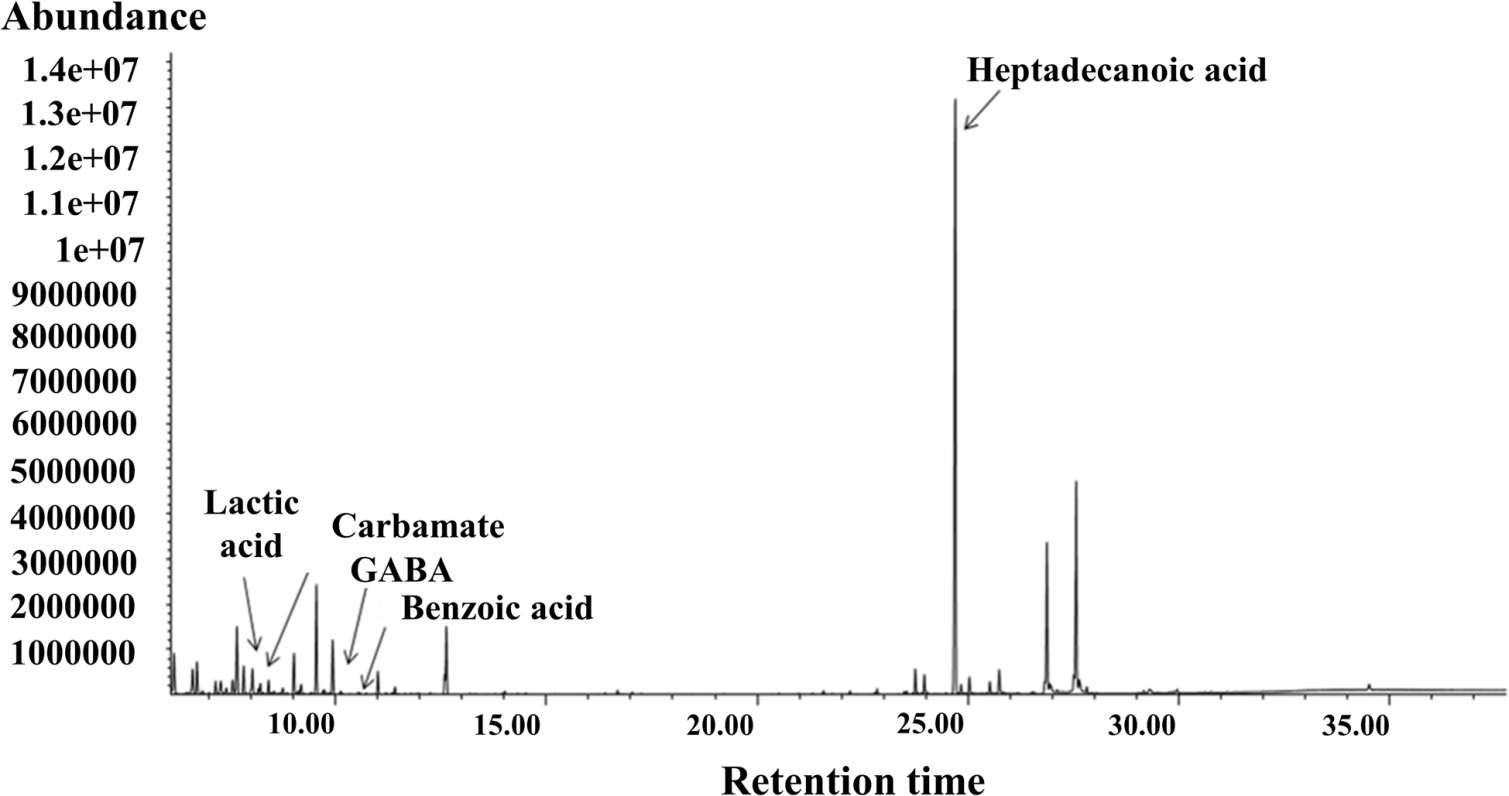
Representative chromatogram of metabolites from spermatozoa bulls. Peaks of GABA, carbamate, benzoic acid, lactic acid, and heptadecanoic acid (internal standard) are indicated

Twenty-two metabolites were structurally identified in the bull spermatozoa, regardless of fertility phenotype of the animals. As the full scan spectra of the metabolites showed consistency in all samples, the retention time, target ion, two quantitative ions, and chemical structure of the derivative product of each metabolite were used for the metabolite identification (Table 1). The NIST Mass Spectral Search Program (NIST/EPA/NIH Mass Spectral Library, Version 2.0) was also employed to identify all peaks in the chromatograms. An example of a chromatogram of bull sperm metabolites is depicted in Fig. 1. In our study, some eluting compounds in GC-MS spectra could not be identified because of the limitation of single quadrupole technology and the lack of spectral information in the current databases.

**Table 1:** Metabolites identified in bull spermatozoa by GC-MS

Compounds	RT	TI	QI 1	QI 2	Mean relative abundance (± SD)	Min/Max values	FC	*t*-test *P*-value
*Amino acids, peptides, and analogs*								
GABA	11.40	147	73	174	7.7^E-03^ ± 8.2^E-04^	3.6^E-03^/0.01	1.61	*0.023*
L-Serine	13.19	147	73	103	6.1^E-04^ ± 1^E-04^	4.6^E-05^/1^E-03^	1.33	0.211
*Carbohydrates and carbohydrate conjugates*								
Glycerol	12.62	147.2	73	205.2	0.09 ± 4.7^E-03^	0.075/0.123	1.04	0.390
*Carboxylic acids and derivatives*								
Acetic acid	13.29	191	73	146.8	8.2^E-04^ ± 1^E-04^	1.2^E-04^/0.002	0.68	0.211
Acetate	10.10	147	73	155	6.0^E-03^ ± 5.9^E-03^	3.1^E-03^/9.1^E-03^	0.81	0.142
*Fatty acids and conjugates*								
Nonanoic acid (C9:0)	13.94	117	73	202	4.5^E-03^ ± 4^E-04^	2^E-03^/6 ^E-03^	1.01	0.246
Azelaic acid (C_9_H_16_O_4_)	20.51	317.2	73	201	0.02 ± 2.4^E-04^	1^E-03^/ 3^E-03^	1.09	0.397
Oleic acid (C18:1 n-9)	27.56	131.2	73	144.2	0.33 ± 0.02	0.256/0.446	0.99	0.481
Oleanitrile (C_18_H_33_N)	23.96	122.2	69.2	55	5.9^E-03^ ± 5.5^E-04^	0.018/0.035	1.05	0.395
Palmitic acid (C16:0)	23.54	313.4	73	117	5.9^E-03^ ± 5.5^E-04^	3^E-03^/ 8^E-03^	0.77	*0.040*
*Inorganic compounds*								
Phosphoric acid	12.58	299	73	357	0.2 ± 0.06	0.065/0.82	0.73	0.228
Borate	6.59	221	73	157	0.071 ± 9.3^E-03^	0.033/0.12	0.91	0.366
Phosphine	9.53	116	73	204	0.14 ± 8.1^E-03^	0.109/0.176	0.93	0.390
*Keto acids and derivatives*								
2-Ketobutyric acid	14.31	202.2	73	111.8	4.5^E-04^ ± 1^E-04^	1.1^E-04^/1 ^E-03^	0.89	0.474
*Organic acids and derivatives*								
Lactic acid	8.20	117	73	147	9.7^E-04^ ± 1^E-03^	3.4^E-03^/0.016	1.59	*0.040*
Oxalic acid	8.16	147	73	133	0.01 ± 4.3^E-04^	9.1^E-03^/0.013	1.02	0.403
Urea	11.83	147	73	189	5.9^E-03^ ± 8.2^E-04^	1.1^E-03^/9.1^E-03^	1.03	0.452
Benzoic acid	11.90	105	77	179	1.2^E-03^ ± 8.6^E-05^	9.9^E-03^/1.9^E-03^	1.46	*0.034*
Carbonate	12.32	191	73	199	5.7^E-04^ ± 1.4^E-04^	3.5^E-07^/0.001	1.00	0.493
Carbamate	7.651 8.019	147 174	73 73	204 198	0.05 ± 0.01 0.14 ± 1^E-03^	0.044/0.057 0.061/0.186	1.08 1.47	0.171 *0.033*
*Steroids and steroid derivatives*								
Cholesterol	34.50	129.2	73	207.2	7.1^E-03^ ± 9.4^E-04^	3.7^E-03^/1.3^E-02^	1.03	0.442

*RT* Retention time, *TI* Target ion, *QI* Quantitate ion, *SD* Standard deviation, *Min* Minimum, *Max* Maximum, and *FC* Fold change. *P*-value is determined by the *t*-test to determine the statistical differences in metabolite abundances between HF and LF groups. *P*-values that are < 0.05 are italicized

The metabolites identified in samples of bovine spermatozoa were categorized into eight major chemical classes, as determined by hierarchical clustering analysis: organic acids/derivatives, fatty acids and conjugates, inorganic acids and derivatives, carboxylic acids and derivatives, amino acids, peptides/analogues, keto acids and derivatives, steroids and derivatives, and carbohydrates/carbohydrate conjugates (Table 1).

**Fig. 2:**
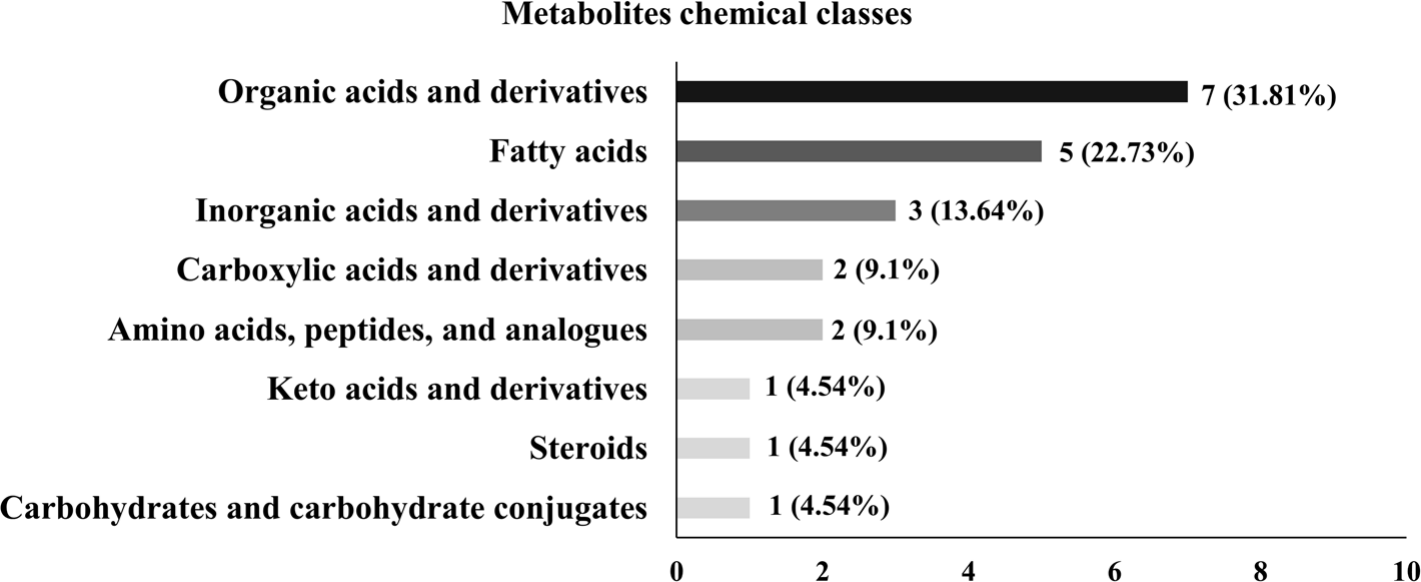
Number of metabolites per chemical class. Metabolites identified in bull spermatozoa according to their chemical classes, defined as amino acids, peptides/analogs, carbohydrates/carbohydrate conjugates, fatty acids, steroids/steroid derivatives, keto acids and derivatives, organic and inorganic compounds, carboxylic acids and derivatives

Organic acids/derivatives were the largest group of metabolites identified in the bull sperm by GC-MS, representing 31.81% of all metabolites in those cell types. Detected organic acids/derivatives were lactic acid (9.7^E-04^ ± 1^E-03^; 3.4^E-03^/0.016), oxalic acid (0.01 ± 4.3^E-04^; 9.1^E-03^/0.013), urea (5.9^E-03^ ± 8.2^E-04^; 1.1^E-03^/9.1^E-03^), benzoic acid (1.2^E-03^ ± 8.6^E-05^; 9.9^E-03^/1.9^E-03^), carbonate (5.7^E-04^ ± 1.4^E-04^; 3.5^E-07^/0.001) as well as carbamate (0.05 ± 0.01; 0.044/0.057) and (0.14 ± 1^E-03^; 0.061/0.186). The second largest group of sperm metabolites covered a remarkable diversity of fatty acids (22.73%), including nonanoic acid (4.5^E-03^ ± 4^E-04^; 2^E-03^/6 ^E-03^), azelaic acid (0.02 ± 2.4^E-04^; 1^E-03^/ 3^E-03^), oleic acid (0.33 ± 0.02; 0.256/0.446), oleanitrile (5.9^E-03^ ± 5.5^E-04^; 0.018/0.035), and palmitic acid (5.9^E-03^ ± 5.5^E-04^; 3^E-03^/8^E-03^). In addition, there were three inorganic compounds, such as phosphoric acid, (0.2 ± 0.06; 0.065/0.82), borate (0.071 ± 9.3^E-03^; 0.033/0.12), and phosphine (0.14 ± 8.1^E-03^; 0.109/0.176), making 13.64% of all metabolites detected by GC-MS in the bull spermatozoa. Carboxylic acids and derivatives (9.1%) including acetic acid (8.2^E-04^ ± 1^E-04^; 1.2^E-04^/0.002) and acetate (6.0^E-03^ ± 5.9^E-03^; 3.1^E-03^/9.1^E-03^) were also identified. Amino acids such as gamma-aminobutyric acid (GABA; 7.7^E-03^ ± 8.2^E-03^; 3.6^E-03^/0.01) and L-serine (6.1^E-04^ ± 1^E-01^; 4.6^E-05^/1^E-03^) comprised 9.1% of the bull sperm metabolite profile. Carbohydrates/carbohydrate conjugates (4.54%) such as glycerol (0.09 ± 4.7^E-03^; 0.075/0.123) as well as steroids and derivatives (4.54%) such as cholesterol (7.1^E-03^ ± 9.4^E-04^; 3.7^E-03^/1.3^E-02^) were also detected. Furthermore, a member of keto acids and derivative class (4.5%), 2-ketobutyric acid (4.5^E-04^ ± 1^E-04^; 1.1^E-04^/1^E-03^), was identified in bull spermatozoa (Table 1 and Fig. 2).

**Fig. 3:**
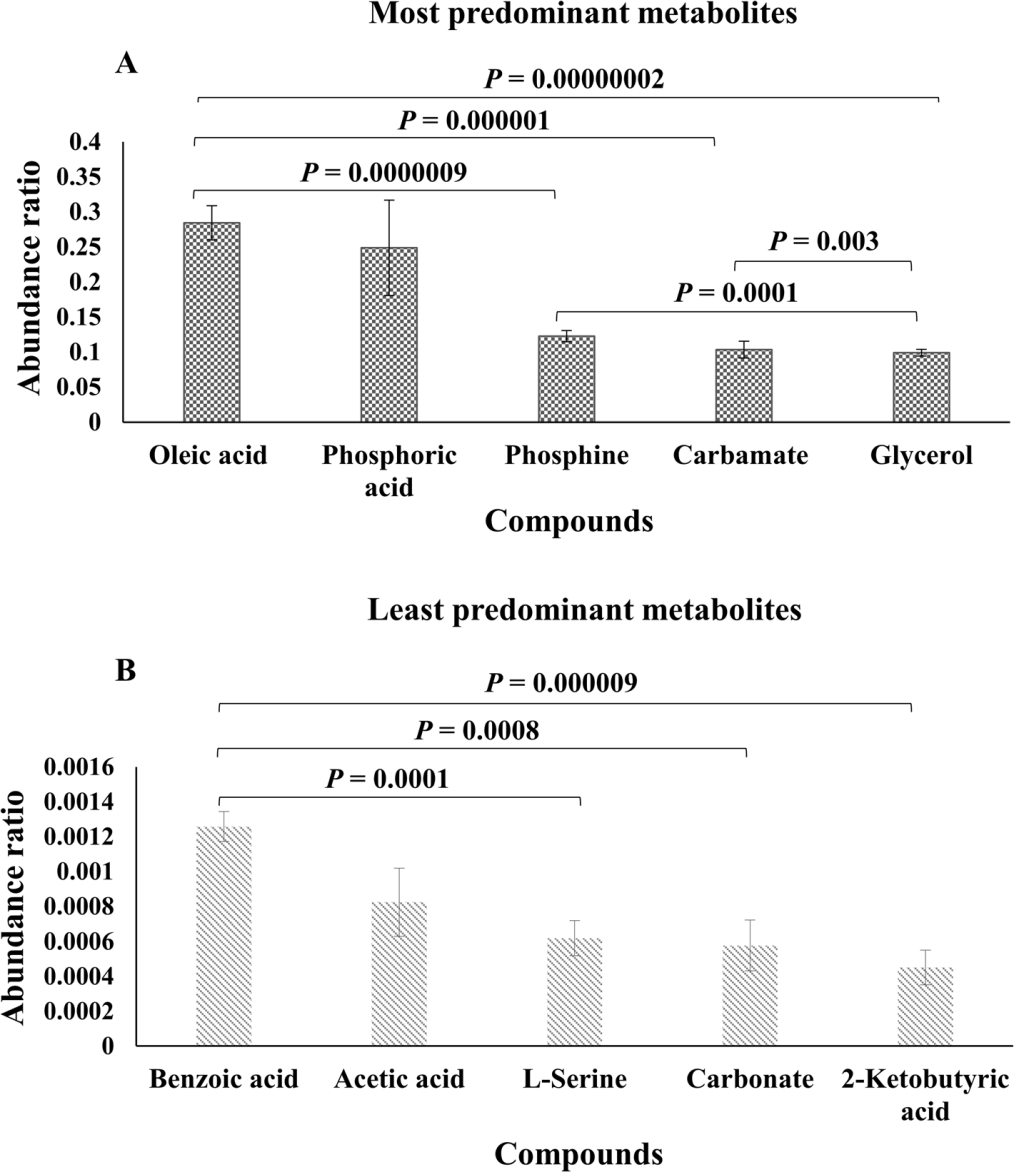
Abundance ratios of the most and least predominant metabolites present in bull spermatozoa. **a** Abundance ratios of the five most abundant metabolites such as oleic acid, phosphoric acid, phosphine, carbamate, and glycerol. **b** Abundance ratios of the five least metabolites identified as benzoic acid, acetic acid, L-serine, carbamate, and 2-ketobutyric acid. The abundance ratio of the metabolites was calculated by dividing the abundance of target ions of metabolites by that of target ion of the internal standard. Error bars represent standard error of the mean. *P* < 0.05 was considered significant

Oleic acid, phosphoric acid, phosphine, carbamate, and glycerol were the most abundant metabolites in bull spermatozoa (Fig. 3a). In contrast, the least abundant metabolites were benzoic acid, acetic acid, L-serine, carbonate, and 2-ketobutyric acid (Fig. 3b). Based on the analysis of variance, the abundance ratio of oleic acid in the bull sperm was greater than those of phosphine (*P* = 0.0000009), carbamate (*P* = 0.000001), and glycerol (*P* = 0.00000002) (Fig. 3a). Moreover, the abundance ratio of phosphine was greater than those of glycerol (*P* = 0.0001), and the abundance ratio of carbamate was higher than those of glycerol (*P* = 0.003). In addition, the relative abundance within the least predominant metabolites had also displayed significant differences. Bull spermatozoa had a greater abundance ratio of benzoic acid as compared with L-serine (*P* = 0.0001), carbonate (*P* = 0.0008), and 2-ketobutyric acid (*P* = 0.000009), as shown in Fig. 3b.

**Fig. 4:**
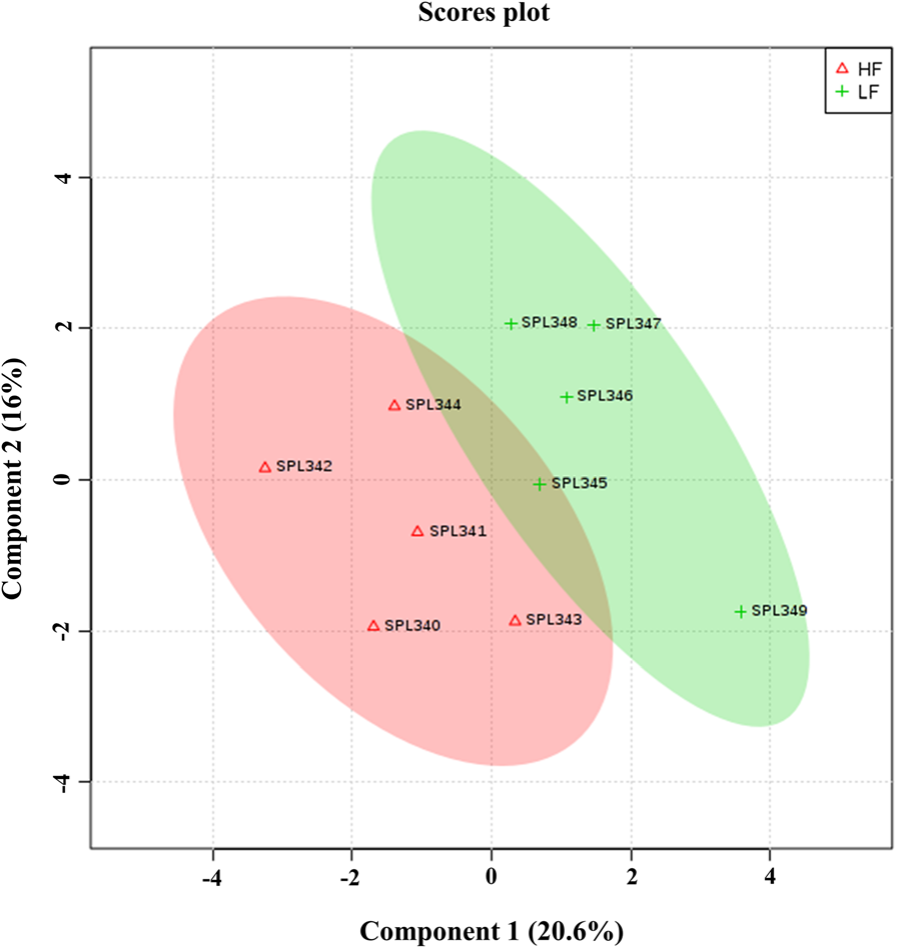
Partial-Least Squares Discriminant Analysis (PLS-DA) scores plot of metabolite profiles generated by GC–MS analysis of bull spermatozoa from high (HF) and low fertility (LF) bulls. Plots showed a clear cluster distinction between HF (*n* = 5) and LF (*n* = 5) groups. Supervised PLS-DA was obtained with 2 components. The explained variances are shown in parentheses

### Associations between sperm metabolites and bull fertility

Partial Least Square-Discriminant Analysis (PLS-DA) analysis was assessed to determine the contribution of metabolites for the separation of high fertility (HF) and low fertility (LF) groups. PLS-DA two-dimensional score plots of sperm metabolites demonstrated that HF and LF phenotypes of bulls were separated from each other in two distinct clusters with a small overlap (Fig. 4). The first two components (1 and 2) explained 20.6 and 16.0% of the variance in the data set, respectively. In addition, the first five components explained 75.6% of the total variance. The metabolites that contributed most to the separation of LF and HF groups were GABA, carbamate, benzoic acid, and lactic acid. The performance characteristics of this multivariate model were R^2^ = 0.428 and Q^2^ = 0.874, respectively.

**Fig. 5:**
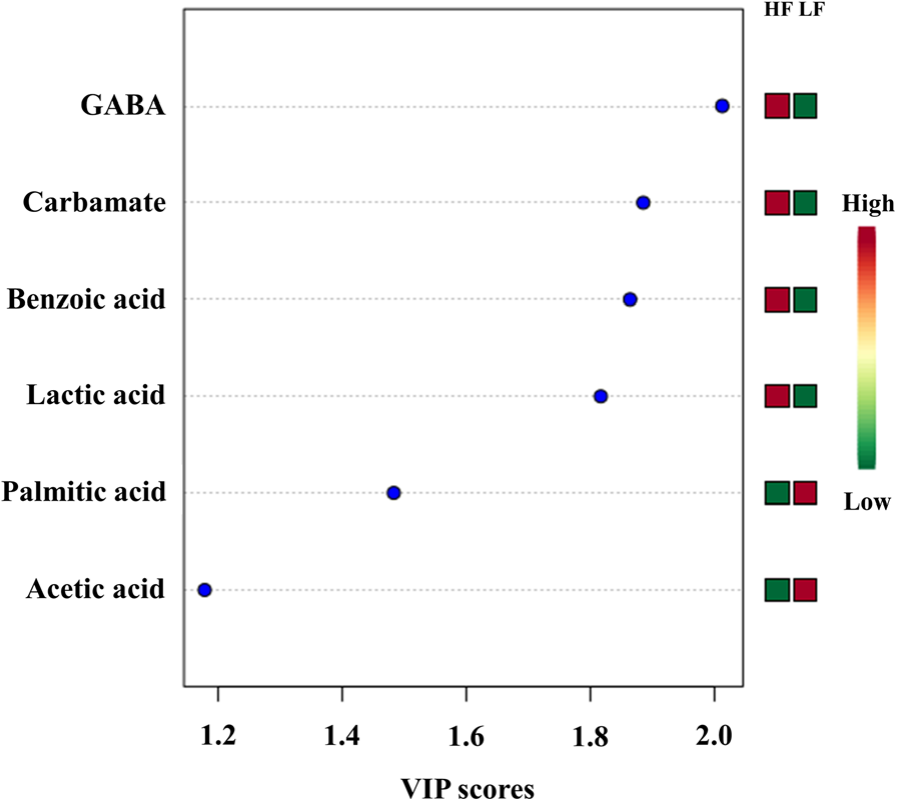
Variable importance in projection (VIP) scores of spermatozoa metabolites in high (HF) and low fertility (LF) bulls. The selected metabolites were those with VIP > 1.5. Heat map with red or green boxes on the right indicates high and low abundance ratio, respectively, of the corresponding metabolite in HF and LF bulls. VIP score was based on the PLS-DA model. GABA (VIP = 2.01), carbamate (VIP = 1.88), benzoic acid (VIP = 1.86), lactic acid (VIP = 1.81), and palmitic acid (VIP = 1.5)

**Fig. 6:**
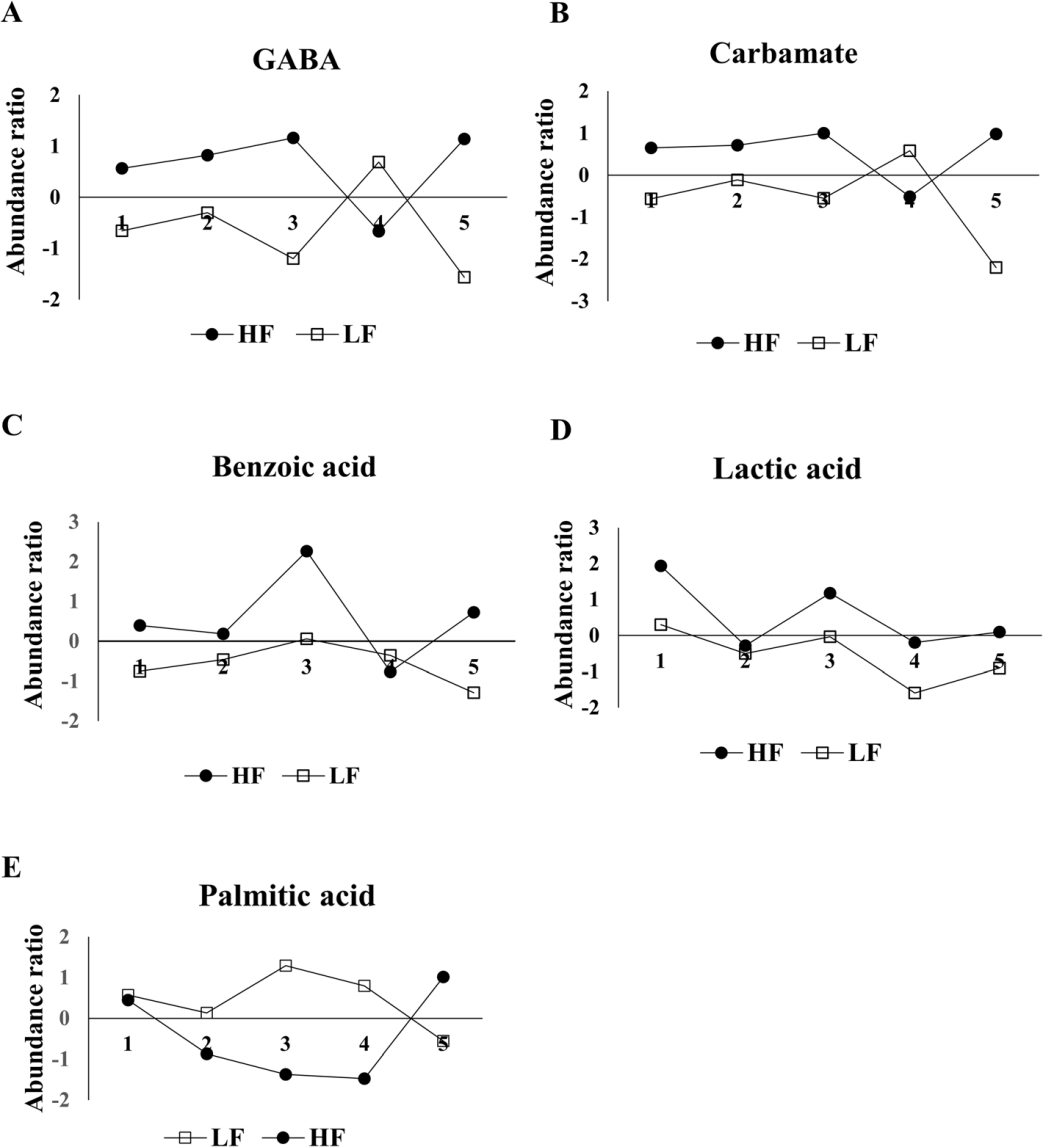
Abundance ratio of five differential metabolites in spermatozoa from high and low fertility bulls. (**a**) GABA (*P* = 0.023), (**b**) Carbamate (*P* = 0.033), (**c**) Benzoic acid (*P* = 0.035), (**d**) Lactic acid (*P* = 0.040), and (**e**) Palmitic acid (*P* = 0.040) were significantly different between high (HF) and low (LF) bulls

The Variable Importance in Projection (VIP) score based on the PLS-DA model represents the potential of the metabolite as a biomarker (Fig. 5) and those variables with VIP score greater than 1.5 were considered important towards the classification model. Five metabolites had VIP scores > 1.5, including GABA (VIP = 2.01), carbamate (VIP = 1.88), benzoic acid (VIP = 1.86), lactic acid (VIP = 1.81), and palmitic acid (VIP = 1.50). Although our results also indicate that differences in metabolite abundance are not consistent between fertility groups (Fig. 6), the abundance ratios of five sperm metabolites were statistically different between LF and HF groups (Fig. 6): GABA, carbamate, benzoic acid, lactic acid, and palmitic acid, as determined by univariate statistical analysis (Table 1).

**Fig. 7:**
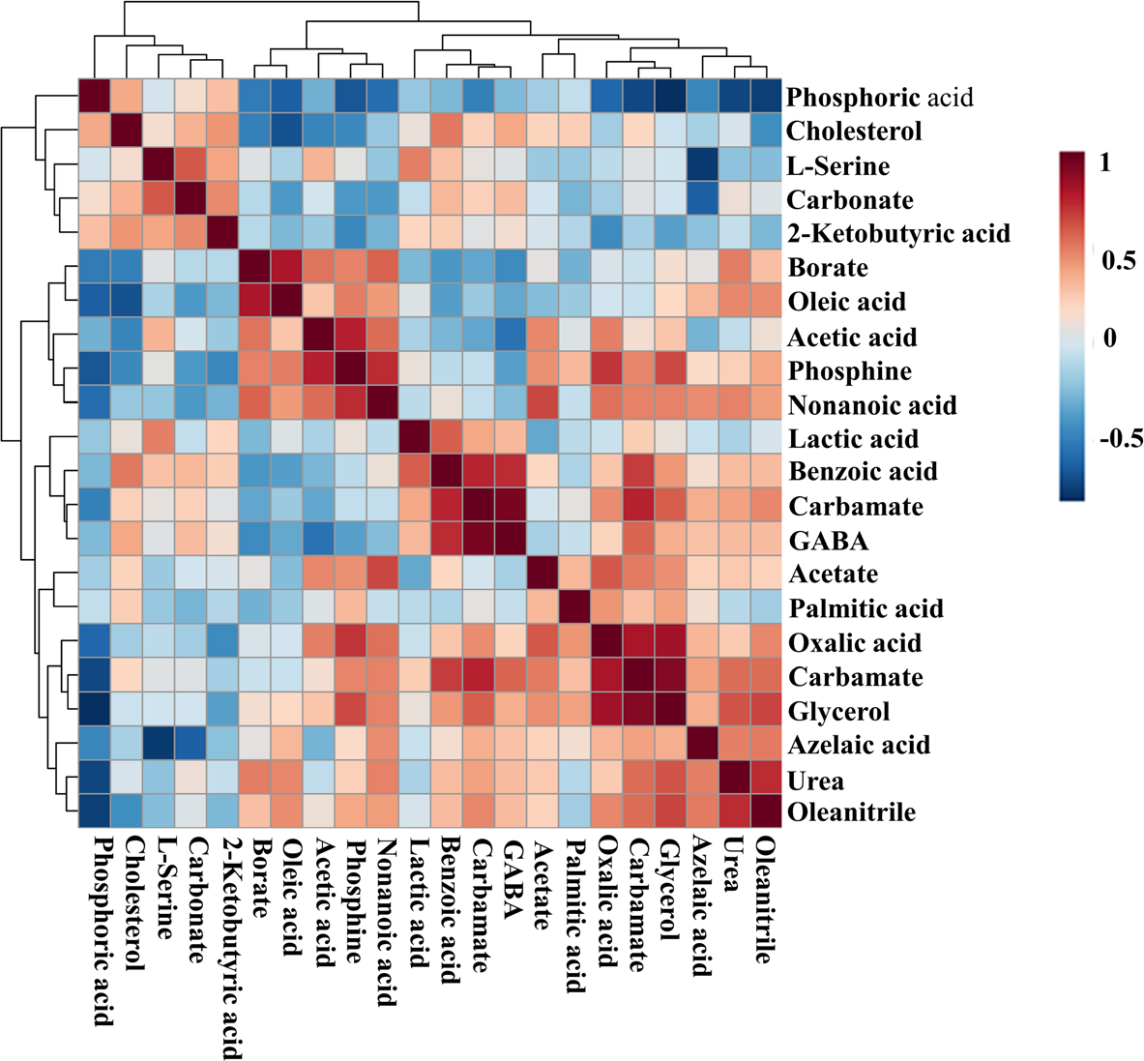
Heatmap visualization of Pearson’s correlations among metabolites present in bull spermatozoa. The scale is based on colors from red (positive) to blue (negative) representing associations between the relative abundance of bull sperm metabolites that related to each other in the groups

The correlation matrix shows positive (red) and negative (blue) associations between the abundance ratios of the metabolites in HF and LF bulls (Fig. 7). Thus, phosphoric acid was inversely associated with oleic acid (r = − 0.64), phosphine (r = − 0.67), oxalic acid (r = − 0.61), glycerol (r = − 0.82), urea (r = − 0.73), and oleanitrile (r = − 0.76). Phosphine had a positive association with acetic acid (r = 0.50), nonanoic acid (r = 0.73), oxalic acid (r = 0.71), and glycerol (r = 0.64). In addition, carbamate was positively correlated with benzoic acid (r = 0.75) and glycerol abundance was related to that of oxalic acid (r = 0.59), carbamate (r = 0.83), urea (r = 0.62) and oleanitrile (r = 0.66). L-serine had positive correlation with carbonate (r = 0.61) and negatively linked to azelaic acid (r = − 0.78) in the bovine sperm. L-serine had also a negative association with azelaic acid (r = − 0.78) whole GABA was positively correlated with carbamate (r = 0.94) and benzoic acid (r = 0.74).

### Diagnosis evaluation of the biomarkers

**Fig. 8:**
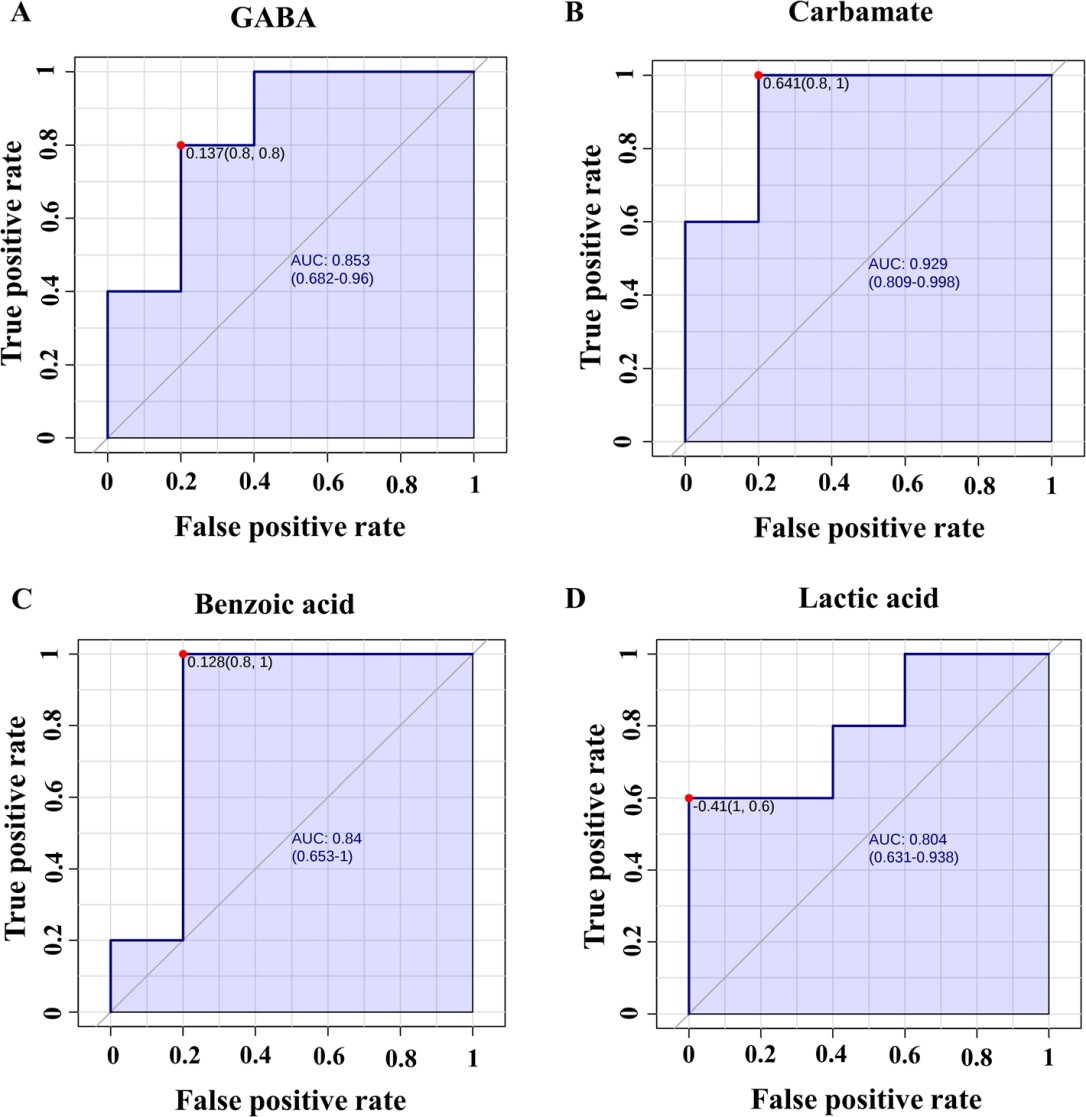
Receiver operating characteristic curves based on PSL-DA analysis models. **a** GABA (AUC: 0.84; *P* = 0.001), **b** Carbamate (AUC: 0.92; *P* = 0.005), **c** Benzoic acid (AUC: 0.84; *P* = 0.006), and **d** Lactic acid (AUC: 0.80; *P* = 0.008). AUC: Area under curves

Multivariate ROC analyses were used to assess the sensitivity and specificity of the potential biomarkers of bull fertility. By analyzing the data, we demonstrated that all the area under the receiver operating characteristic (ROC) curve (AUC) of the sperm metabolites ranged from 0.52 to 0.92. Metabolites with an AUC > 80% were carbamate (AUC = 0.92; *P* = 0.005), GABA (AUC = 0.84; *P* = 0.001), benzoic acid (AUC = 0.84; *P* = 0.006), and lactic acid (AUC = 0.80; *P* = 0.008; Fig. 8).

### Functional biochemical pathway analysis

**Fig. 9:**
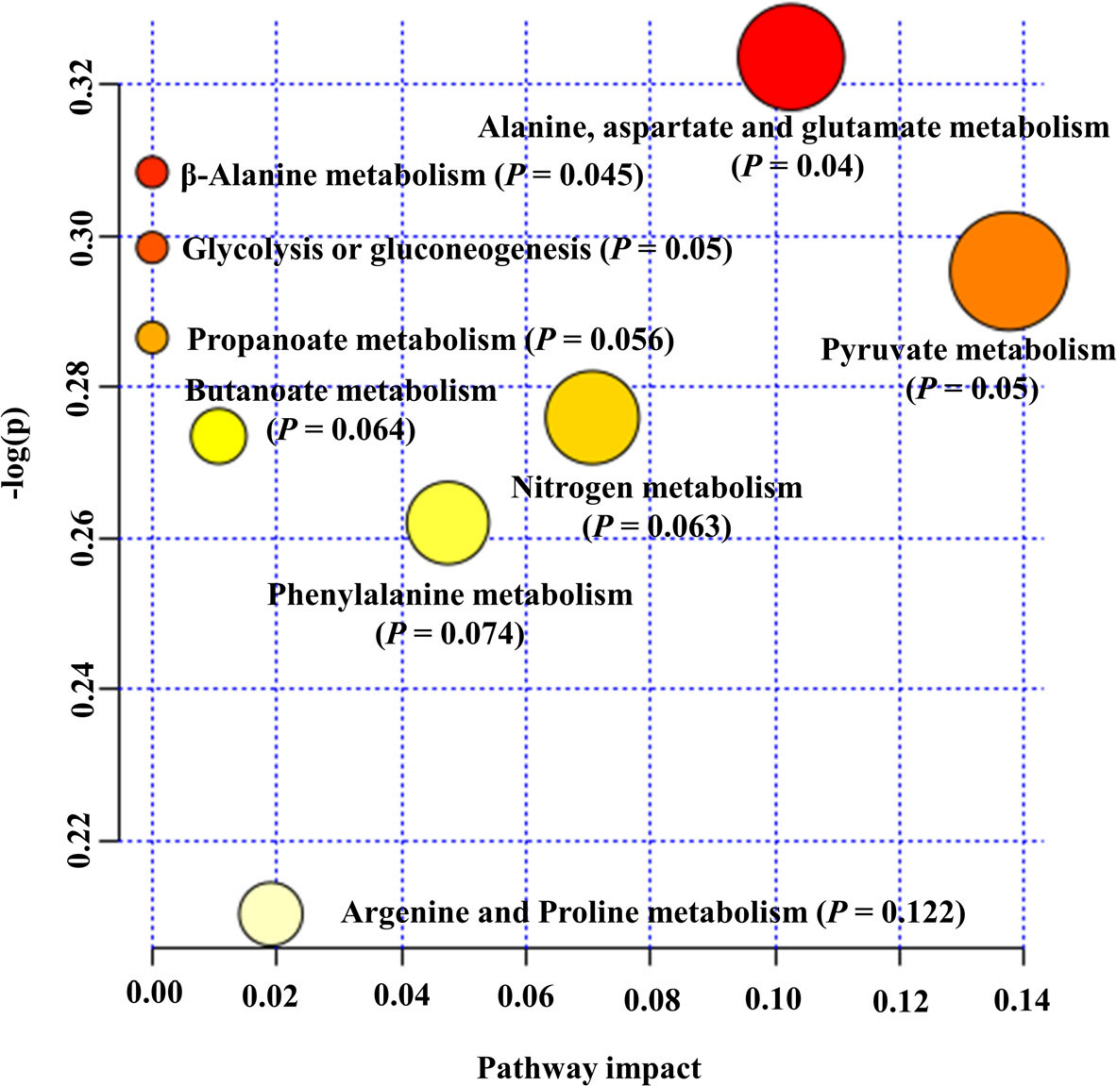
Pathway analysis of differentially present metabolites in bull as determined by MetaboAnalyst 4.0. Each point represents one metabolic pathway; the size of the dot is in positive correlation with the impaction of the metabolic pathway

Metabolic pathway analysis was performed to evaluate the most relevant pathways associated with differential metabolites in the sperm of HF and LF bulls. The differential metabolites encompass four biochemical pathways, which may reveal the metabolic mechanisms within spermatozoa that might affect fertility. The metabolic pathways were alanine, aspartate and glutamate metabolism (*P* = 0.04), β-alanine metabolism (*P* = 0.045), glycolysis or gluconeogenesis (*P* = 0.05), and pyruvate metabolism (*P* = 0.05), as shown in Fig. 9.

## Discussion

Characterization of the sperm metabolic signatures is a powerful approach that can potentially lead to the development of biomarkers for male fertility. In the present study, we investigated the metabolic profiles of spermatozoa from bulls with high vs. low fertility status using non-targeted metabolomics as well as statistical and bioinformatics tools. Results presented here are an important foundation to further understand the mechanisms by which metabolites of spermatozoa may affect fertility outcomes and to help to predict the fertilizing potential of sires.

Metabolites play key roles in sperm physiology and are related to differences in male fertility phenotypes [31–33, 37]. Recently, NMR- and GC-MS-based studies showed that pathways for nucleoside, amino acid, and energy metabolism were disturbed in asthenozoospermic men [32], and that metabolites found in human spermatozoa are associated with semen parameters [37]. In this study, our analyses by GC-MS revealed that the majority of metabolites of bovine sperm are organic acids and derivatives, followed by a group of fatty acids and conjugates. Organic acids are produced by the breakdown of amino acids and fatty acids, and the degradation of such metabolites generates energy substrates for tricarboxylic acid (TCA) cycle and respiratory chain [38]. The presence of organic acid suggests that bull spermatozoa have active energy metabolism [8, 39, 40]. In addition, organic acids play crucial roles during anabolism by providing C-atom backbones [38]. Studies have previously reported the presence of organic acids in human spermatozoa [31, 32] and seminal plasma of bulls [30] and humans [27, 41, 42]. Fatty acids, in turn, are involved in the structural organization of the sperm membranes, energy metabolism, and signaling molecules [12, 43, 44]. The enzymatic machinery for beta-oxidation is present in human spermatozoa [45, 46], suggesting that sperm may obtain energy also through the oxidation of fatty acids [47]. Several types of fatty acids have also been reported in seminal plasma of humans and bulls [27, 30, 31].

The most predominant metabolites of the bull spermatozoa were oleic acid, phosphoric acid, phosphine, carbamate, and glycerol; whereas benzoic acid, acetic acid, L-serine, carbonate, and 2 ketobutyric acid were among the least abundant metabolites. Oleic acid (C18:1 n-9) is the most abundant monounsaturated fatty acids of the plasma membrane of ejaculated stallion [48], boar [49], and ram spermatozoa [50]. Oleic acid is negatively linked to sperm motility and concentration in humans [51–53]. In addition, high levels of oleic acid have been reported to increase lipid oxidation [53], leading to disorders in sperm membrane metabolism in men [27]. On the other hand, addition of oleic acid maintains bull sperm viability and lowers the production of reactive oxygen species (ROS) in vitro [54]. The high content of oleic acid in bovine sperm suggests its contribution to reduce ROS production [54] and to generate energy for sperm hyperactivation [55]. Phosphoric acid was the second most abundant metabolite in bull spermatozoa. In normal sperm cells, phosphoric acid is produced by the breakdown of ATP in a reaction catalyzed by inorganic pyrophosphatase (PPA1) [56]. PPA1 catalyzes the hydrolysis of one molecule of inorganic pyrophosphate (PPi) to two molecules of phosphoric acid, leading to the release of energy in form of adenosine triphosphate (ATP). The transport of PPi from spermatozoa to the seminal plasma may be regulated by a transmembrane protein, called progressive ankylosis protein (ANKH). In mammals, PPA1 is present in the post-acrosomal sheath of the sperm head and in the distal part of sperm acrosome. The energy produced from the conversion of PPi to phosphoric acid could be used for sperm motility and for acrosomal function during sperm-zona penetration [57, 58]. In addition, inorganic phosphate resulted from the hydrolysis of ATP positively affect both motility and fertilizing capacity of human sperm [59]. Thus, high levels of inorganic phosphate in bull spermatozoa may be required to maintain sperm motility status and to achieve normal fertility.

Our GC-MS-based analyses indicated that carbamate is the fourth most abundant metabolite of the bovine spermatozoa with the second highest VIP score. Carbamate was first reported in seminal plasma from healthy and asthenozoospermic men [27]. However, it is new the description of carbamate in bull spermatozoa as well as its higher abundance in sperm from HF bulls. Endogenous carbamate is generated by the interaction of cellular carbon dioxide (CO_2_) with an NH_2_ group of primary and secondary amines [60, 61] when the concentration of CO_2_ increases [62]. The formation of carbamate influences the function of hemoglobin as well [63]. Although the importance of carbamate in sperm physiology is unknown, we can speculate that the spermatozoon, like other cells, employs several mechanisms to maintain the cell pH [64]. Therefore, carbamate formation might be an important mechanism by which spermatozoa regulate their intracellular pH.

The principal inhibitory neurotransmitter in the adult brain, GABA, was found with greater abundance in spermatozoa from HF sires and it had the highest VIP score. The key enzyme in the synthesis of the GABA, glutamate decarboxylase, and GABA_A_/GABA_B_ receptors were previously identified in human spermatozoa [65]. GABA has been also detected in seminal plasma and spermatozoa from humans [66], as well as in seminal plasma of bulls [30]. GABA induces capacitation of spermatozoa from bulls [67, 68], rats [69], and rams [70] and acrosome reaction of bovine sperm [68]. The high abundance of GABA in the sperm of HF bulls may be explained by the roles described above, and in sperm hyperactivation [71]. In the present work, abundance ratios of GABA and carbamate were positively associated, and this may be related to the fact that carbamate modulates GABA_A_ receptor [72]. Moreover, a positive link was also found for GABA and benzoic acid in bull spermatozoa. A previous in vitro study reported that benzoic acid increases efflux of glutamate [73] and levels of benzoic acid may regulate sperm function since GABA is formed by decarboxylation of L-glutamate [74]. The abundance ratios of benzoic acid were increased in spermatozoa of HF bulls as compared to LH sires. The presence of benzoic acid was reported in seminal plasma of bulls [30] and asthenozoospermic and normozoospermic men [27] as well as in spermatozoa from asthenozoospermic and normozoospermic men [32]. A recent study reported a positive correlation between the abundance ratios of benzoic acid and sperm counts in rats [75], suggesting that benzoic acid plays a role in male fertility.

Lactate is an important energy source for bull, human, stallion, and boar spermatozoa [8, 46]. The production of lactate by the bull sperm occurs mainly through glycolysis and mitochondrial oxidative phosphorylation (OxPhos) [8, 45, 47, 76]. Multivariate statistical analysis conducted in the present study demonstrated that lactate was one of the metabolites contributing to fertility phenotype with the fourth highest VIP associated with HF bulls. Greater lactate abundance in HF bulls suggests that these animals utilize anaerobic glycolysis more efficiently than LF sires [8, 77]. It is well-known that sperm mitochondria compensate for decreased energy production by increasing lactate yield under hypoxia. The efficient glycolysis is dependent on either endogenous or exogenous pyruvate, which indirectly feeds the accelerated glycolysis with nicotinamide adenine dinucleotide (NAD^+^) through the lactate dehydrogenase-mediated conversion of pyruvate to lactate [8, 76]. The oxidation of NAD^+^ in the electron transport chain generates the ATP molecules by oxidative phosphorylation [8]. Thus, when high energy is required for sperm motility and other events, spermatozoa efficiently metabolize glycolysable substrates to yield ATP [8, 46]. In fact, the inhibition of lactate dehydrogenase blocks sperm capacitation in bulls [78], humans [79], mice [80], and goats [81]. Therefore, the level of lactate in sperm could be considered as an early indicator of bull fertility [82].

Palmitic acid (C16:0), another metabolite found in bull spermatozoa, had the fifth highest VIP score associated with LF animals. This is consistent with previous studies showing increased levels of palmitic acid in infertile men [27, 83, 84] and in asthenozoospermic semen as compared to normozoospermic ones [85]. Another study reports that high palmitic acid in seminal plasma from asthenozoospermic men indicates a disorder in sperm membrane metabolism [27].

The importance of lipid metabolism for the production of energy for spermatozoa has been discussed in previous studies [43]. Our analytical methods allowed the detection of considerable amounts of nonanoic acid and azelaic acid in bull spermatozoa. Nonanoic acid (C9:0), also known as pelargonic, is a 9-carbon chain fatty acid, and it was previously reported in goat epididymal sperm membrane [86] and mouse epididymal fluid [87]. The importance of nonanoic acid for sperm physiology is still unknown, but it is possible that it contributes to sperm maturation [87]. In addition, OR51E1, a known receptor of nonanoic acid, was detected in the acrosomal cap of human spermatozoa [88, 89], and activation of OR51E1 with nonanoic acid led to the phosphorylation of various protein kinases [90]. Also, OR51E1 level decreased upon acrosomal exocytosis [91], and such results suggest that nonanoic acid is involved in acrosome reaction, possibly by triggering protein tyrosine phosphorylation during sperm capacitation.

The present study reported for the first time the presence of azelaic acid (nonanedioic acid; C9H16O4) in bull spermatozoa. Azelaic acid is a nine-carbon saturated aliphatic dicarboxylic acid, and it has been reported in testes of rats [92]. This metabolite was also found in mouse [93] and human spermatozoa [31]. Azelaic acid is the end product of linoleic acid peroxidation [94] and acts as a ROS scavenger [95], protecting spermatozoa. Moreover, studies mention additional roles for azelaic acid including inhibition of tyrosinases [96, 97], mitochondrial enzymes [98], anaerobic glycolysis [98], mitochondrial oxidoreductase activity, and DNA synthesis [99]. A study showed evidence that the incubation of mouse sperm in fructose-containing media resulted in a high concentration of azelaic acid in sperm when compared with glucose-containing media [93]. Given that azelaic acid modulates the activity of glycolytic key enzymes [100], we hypothesize that this metabolite is essential for energy metabolism of the sperm cells.

We also evaluated the metabolic pathways of certain molecules and their potential contributions to male fertility. There were four significant pathways associated with differential sperm metabolites including alanine, aspartate and glutamate metabolism, β-alanine metabolism, glycolysis or gluconeogenesis, and pyruvate metabolism. Alanine, aspartate, and glutamate are linked to amino acid metabolism. As amino acids play key roles in multiple cellular processes, they influence the metabolic activity of the spermatozoa [101]. The GABA is involved in sperm motility, acrosome reaction, and fertilization in human spermatozoa [102]. Thus, we assume that as spermatozoa of HF animals have more GABA their fertility rate increases. Another amino acid-related pathway identified in bull sperm was β-alanine metabolism. β-alanine is structurally intermediate between alpha-amino acid (glycine, glutamate) and GABA neurotransmitters [103], and β-alanine is a ubiquitous amino acid correlated with the TCA cycle. In fact, both TCA intermediates and amino acids have been found as part of the metabolomic profile of bull seminal plasma, as recently described by Velho et al. [30]. The glycolysis consists of a series of biochemical reactions to generate energy in the form of ATP [77]. Glycolytic metabolite such as lactate was significantly elevated in spermatozoa from HF bulls as compared to LF animals, suggesting that the maintenance of intracellular energy status is essential for sperm function. Considering the pathways analyzed in the present study, pyruvate metabolism is crucial for understanding the contributions of OxPhos for the fertilizing capacity of spermatozoa [45]. Bull spermatozoa also rely on OxPhos to maintain sperm functions [77]. A recent study showed that pyruvate is the most important source of energy for stallion sperm [8] and that the impairment of sperm mitochondrial ultrastructure may affect male fertility [104].

Although the sample size represents a limitation of the present study, the sensitivity of the GC-MS approach, together with the bioinformatic tools, enabled us to construct a metabolomics analytical model of sperm from bulls with different fertility phenotypes. Metabolites with different abundances in bulls of high and low fertility (GABA, carbamate, benzoic acid, lactic acid, and palmitic acid) are potential biomarkers of bull fertility.

## Conclusions

The metabolomic signatures of bull spermatozoa advance our current understanding of the multifactorial and complex processes related to the physiology of male fertility. The present study uncovered vital pieces of information about sperm metabolites for diagnosing male fertility. In addition, because of the strong similarities in physiology and genetics between cattle and other mammals, including humans and endangered mammals, the knowledge generated in the present investigation can be applied to enhance reproductive biotechnology of other species.

## Methods

### Study design

Metabolomic analysis of bull spermatozoa with two distinct and reliable fertility phenotypic scores was performed by GC-MS. Univariate and multivariate statistical models were employed to identify key differences between the two groups, HF (*n* = 5) and LF (n = 5) bulls. Statistical and bioinformatics tools were also used to identify potential biomarkers of bull fertility.

### Determination of fertility phenotypes of dairy bulls

In the current study, the field fertility data were collected for the evaluation of fertility scores of mature Holstein bulls (Table 2), as previously described by Peddinti et al. [105]. Fertility data were obtained from the Alta Advantage Program (Alta Genetics, Inc., Watertown, WI, USA), which periodically updates results from AI in the partnering herds [105]. The conception rates were confirmed in the field by either ultrasound or veterinary palpation. The method used for the calculation of bull fertility was similar to the one employed in previous investigations [17–19, 29]. Factors that influenced the fertility of sires such as environmental and herd management were adjusted using a model previously described [106, 107]. The average conception of breeding records and conception rates was calculated using the Probit F90 software [108]. The bulls were selected with conception rates of two standard deviations above and below the average conception rates of the population of sires available in Alta Genetics database. When bulls had percent differences in their conception rates above average, we defined them as “HF”; in contrast, if sires had percent differences in their conception rates below average, we designated them as “LF” (Table 2).

**Table 2:** Fertility scores of mature Holstein bulls. High fertility (HF) bulls were designed from 1 to 5 and bulls 5 to 10 were grouped as low fertility (LF). Fertility score of each bull was expressed as the percent difference of its conception rate from the average conception rate of all bulls. Probit.F90 software was used to estimate bull fertility

Bull #	Fertility status	Number of breedings	Conception rates difference from average (%)	Std of difference	Conception rates (%)
1	HF	5293	5.42	2.02	45.3
2	HF	825	5.1	1.90	40.4
3	HF	2032	4.8	1.79	40.3
4	HF	2487	3.59	1.34	45.7
5	HF	5751	3.56	1.33	39.8
6	LF	1604	−3.75	−1.40	35.7
7	LF	2276	−4.06	−1.52	37.8
8	LF	967	−4.49	−1.68	34.4
9	LF	5603	−6.76	−2.52	34.6
10	LF	674	−10.61	−3.96	23.3

### Sperm collection and preparation

Semen samples from 10 mature Holstein bulls with different fertility scores were provided by Alta Genetics (Watertown, WI, USA). All animals were raised under the same management conditions and received the same nutrition. Ten ejaculates, one per bull, were collected using an artificial vagina and spermatozoa were separated from seminal plasma by centrifugation (700 g, 4 °C, 10 min). Then, the pellet containing spermatozoa was washed twice (700 g; 4 °C; 15 min.) with cold phosphate-buffered saline (PBS) and further aliquoted (100 μl) into a new 2 ml Cryotube® (Sigma-Aldrich, St Louis, MO, USA). Following the second centrifugation, spermatozoa were snap-frozen in liquid nitrogen and transported to Mississippi State University (MSU). At MSU, snap-frozen spermatozoa were stored at − 80 °C until preparation for GC-MS analyses. The metabolomic profiles of the seminal plasma obtained from the same bull semen samples have been reported in our previous publication [30].

### Chemicals and materials

GC-MS grade chemicals and solvents were all purchased from Sigma Aldrich in the highest purity available. Phosphate-buffered solution (137 mM NaCl, 2.7 mM KCl, 8 mM Na_2_HPO_4_, and 2 mM KH_2_PO_4_; pH 7.4) was purchased from Thermo Fisher Scientific (Waltham, MA, USA). Amber glass vials (2 ml) with 300 μl fixed insert were obtained from Agilent Technologies Inc. (Santa Clara, CA, USA).

### Metabolite extraction

**Fig. 10:**
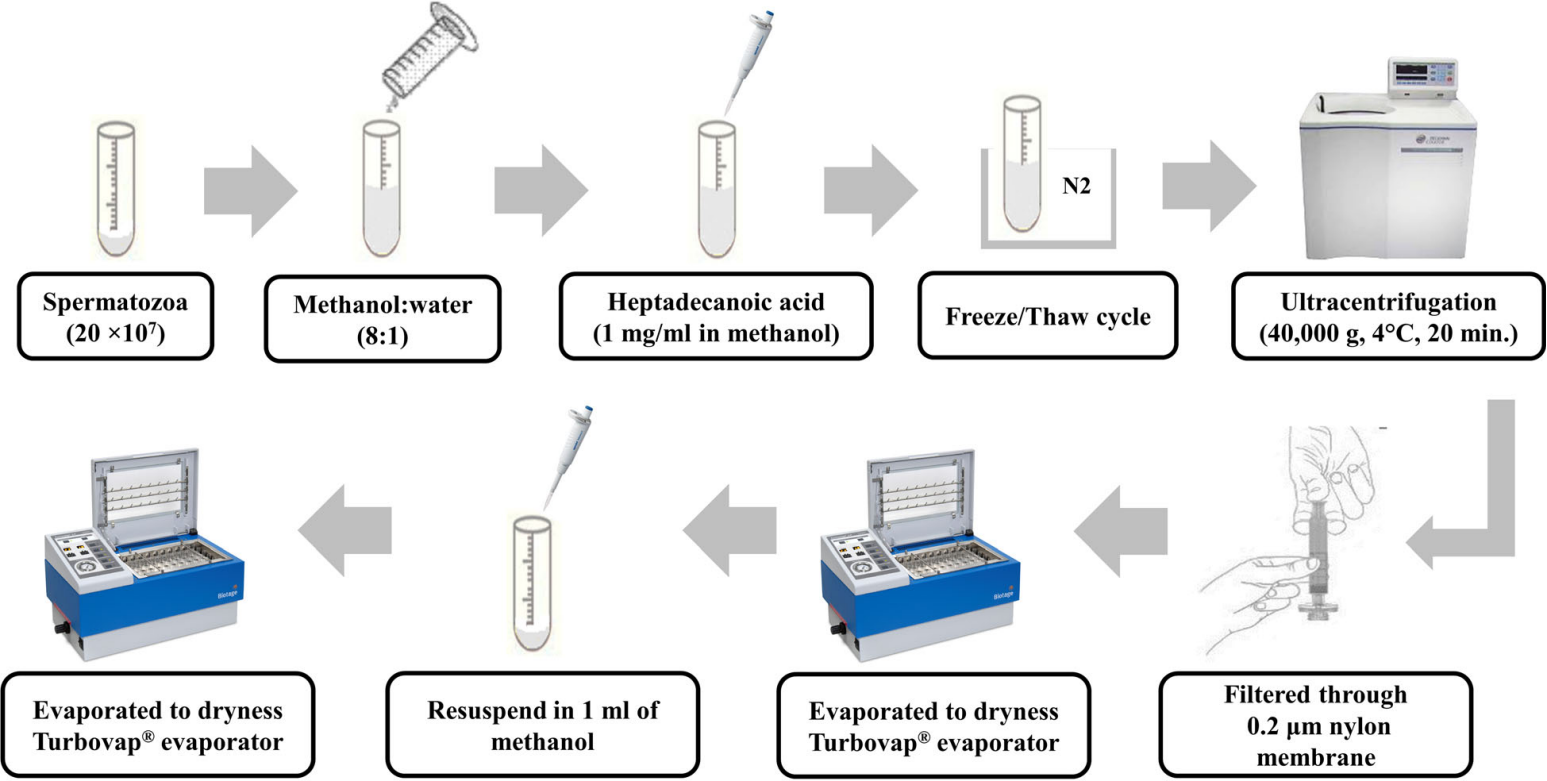
Overview of extraction steps used for the preparation of sperm samples for GC-MS analysis. Drawings are not to scale, see Materials and Methods section for full details

Sperm metabolites were isolated from bull spermatozoa as previously described by Paiva et al. [31], with modifications and a schematic overview of the sperm metabolite extraction is presented in Fig. 10. In summary, snap-frozen sperm (2 × 10^7^ cells) were thawed in a water bath at 37 °C for 30 s. The thawed spermatozoa were suspended in a mixture of 8 ml of methanol and 1 ml of ultrapure water, followed by addition of 150 μl of heptadecanoic acid (1 mg/ml in methanol) as the internal standard. In addition, sperm suspension was subjected to five freeze/thaw cycles. Each cycle consisted of freezing sperm cells in liquid nitrogen vapor for 30 min. and subsequent thawing at room temperature for 30 min. Following freeze/thaw cycles, the cell suspension was sonicated in an ultrasonic bath at 25 °C for 30 min. at 120 W and 40 kHz (Fisher Scientific™ CPXH5 Series Ultrasonic Baths; Pittsburgh, PA, USA), followed by ultracentrifugation (40,000 g, 4 °C, 20 min.) using an OptimaTM L-90 k and Type 70 Ti Rotor (BECKMAN COULTER Life Sciences, Brea, California, USA). The supernatant was filtered through a 0.2 μm nylon membrane (Fisher Scientific, Lenexa, KS, USA) and the filtrate was evaporated under a stream of high-purity nitrogen gas (TurboVap® LV evaporator; Biotage, Charlotte, NC, USA) at 40 °C. An aliquot of methanol (1 ml) was added to dissolve metabolites. The metabolite extracts were subsequently transferred into a 2 ml amber vial and evaporated to dryness again with high-purity nitrogen gas at 40 °C.

The dried extracts were resuspended in 50 μl of methoxyamine hydrochloride (20 mg/ml in pyridine) vortexed vigorously for 1 min, and further heated in a water bath at 70 °C for 1 h. The samples were then derivatized by adding 100 μl N, O-Bis(trimethylsilyl)trifluoroacetamide with 1% trimethylchlorosilane (BSTFA + 1% TMCS) and heated in a water bath at 70 °C for 1 h. The final derivatives were transferred into a new 2 ml amber glass vial with a 300 μl fixed insert for GC-MS analysis. A quality-control sample for the experiment was prepared by pooling equal volumes of sperm extract samples to ensure that the detection of metabolites was consistent.

### Gas chromatography-mass spectrometry (GC-MS) analysis

Screening of the untargeted sperm metabolites was performed using GC-MS as previously described by Velho et al. [30], with modifications. Briefly, metabolite derivatives were separated and detected using an Agilent 7890A GC System coupled to an Agilent 5975C inert XL MSD with triple-axis mass detector, an Agilent 7693 Series Autosampler, and a DB-5MS capillary column (30 m × 0.25 mm i.d. × 0.25 μm film thickness; Agilent Technologies). An aliquot of the derivatized mixture (1 μl) was injected into the inlet heated at 270 °C with a 1:10 split ratio. Standard septum purge was carried out after sample injection at 3 ml/min, and helium carrier gas was at 1 ml/min constant flow rate. Transfer line, ion source, and quadrupole were heated at 260 °C, 200 °C, and 150 °C, respectively. The oven was programmed initially at 80 °C for 2 min., followed by 10 °C/min., ramped up to 180 °C, 5 °C/min. to 240 °C, 20 °C/min. to 290 °C, and 10 min. Maintenance at 290 °C. Ionization was performed in an electron impact mode at 70 eV. Masses were scanned for a full spectrum from m/z 30 to 600 at 10,000 amu/s and 20 scans/s (m/z 0.2 step size). The solvent delay time was 5 min.

### Data processing, calculations, and statistical analysis

Sperm metabolites were identified by their retention time as well as one target and two quantitative ions, in comparison with mass spectra of authentic standards and mass spectra in the NIST mass spectral library. Abundances of the target ions of metabolites were divided by the abundance of target ion of the internal standard (heptadecanoic acid), and the ratios were used for statistical analysis. Identified compounds were categorized based on their chemical classifications using Human Metabolome Database version 3.6 (HMDB; www.hmdb.ca/) [109, 110]. Statistical analysis was carried out using MetaboAnalyst 4.0 Web service (http://www.metaboanalyst.ca). MetaboAnalyst is a comprehensive web-based tool designed to help users easily perform metabolomic data analysis, visualization, and functional interpretation [111]. Sum and auto-scaling normalized each compound. Univariate analysis (*t*-test) was used to determine if differences in metabolite abundances in spermatozoa of HF and LF sires were significantly different.

Multivariate analysis was applied to provide additional information for the interpretation of the data. The PLS-DA defined the separation metabolome of sperm from HF and LF bulls. Potential biomarkers were identified according to the significance of their contributions to variable classification in the PLS-DA model, which was determined by the VIP score. The VIP score summarizes the contribution that a variable makes to the model, and it is calculated as the weighted sum of the squared correlations between the original variable and the PLS-DA components. The weights correspond to the percentage variation explained by the PLS-DA component in the model. The number of terms in the sum depends on the number of PLS-DA components found to be significant in distinguishing the classes. In the present study, we considered metabolites with VIP > 1.5 as potential biomarkers associated with bull fertility. The ROC analysis was applied to examine the specificity and sensitivity of the biomarkers. The area under the ROC curve was calculated to assess the effectiveness of the potential biomarkers. A guide for assessing the performance of metabolites as a biomarker based on its AUC is as follows: AUC of 0.9 to 1.0 = excellent, 0.8 to 0.9 = good, 0.7 to 0.8 = fair, 0.6 to 0.7 = poor, and 0.5 to 0.6 = fail [112]. Pearson’s method was used to analyze the correlation between metabolites. Significance for statistical analyses was set at 0.05.

### Functional biochemical pathway analysis

Differential metabolites were also evaluated by using metabolic pathway analysis (MetPA) [113, 114]. For this analysis, we uploaded the differential metabolites selecting the ‘*Bos taurus*’ library. The default ‘hypergeometric test’ and ‘Relative Betweenness Centrality’ for pathway enrichment and pathway topological analyses, respectively, were selected. Kyoto Encyclopedia of Genes and Genomes (KEGG) metabolic pathway was also employed. All matched pathways were visualized by plotting the −log(p) values from pathway enrichment analysis on Y-axis and pathway impact values from pathway topology analysis on X-axis [114]. The node color was based on its *p*-value and the node radius was associated with their pathway impact values. Metabolic pathways with p-value < 0.05 and false discover rate values of 0.7 were screened as pathways of interest.

## Data Availability

Critical data stemming from this study are include in the manuscript. Additional datasets used and/or analyzed during the current study are available from the corresponding author based on reasonable requests.

## Abbreviations

AI: Artificial insemination
ANKH: Progressive ankylosis protein
ATP: Adenosine triphosphate
AUC: Area under the curve
BSTFA: O-Bis(trimethylsilyl)trifluoroacetamide
CO_2_: Carbon dioxide
GABA: Gamma-aminobutyric acid
GC-MS: Gas chromatography-mass spectroscopy
HF: High fertility
HMDB: Human Metabolome Database
KEGG: Encyclopedia of Genes and Genomes
LF: Low fertility
MetPA: Metabolic pathway analysis
NAD^+^: Nicotinamide adenine dinucleotide
OxPhos: Mitochondrial oxidative phosphorylation
PBS: Phosphate buffered saline
PLS-DA: Partial least squares discriminant Analysis
PPA1: Inorganic pyrophosphatase
PPi: Inorganic pyrophosphate
ROC: Receiver operating characteristic
ROS: Reactive oxygen species
TCA: Tricarboxylic acid
TMCS: Trimethylchlorosilane
VIP: Variable importance in projection

## References

[1] Kaya A, Memili E (2016). Sperm macromolecules associated with bull fertility. Anim Reprod Sci.

[2] Amann RP, DeJarnette JM (2012). Impact of genomic selection of AI dairy sires on their likely utilization and methods to estimate fertility: a paradigm shift. Theriogenology.

[3] Rutten CJ, Steeneveld W, Vernooij JCM, Huijps K, Nielen M, Hogeveen H (2016). A prognostic model to predict the success of artificial insemination in dairy cows based on readily available data. J Dairy Sci.

[4] Santos JE, Thatcher WW, Chebel RC, Cerri RL, Galvao KN (2004). The effect of embryonic death rates in cattle on the efficacy of estrus synchronization programs. Anim Reprod Sci.

[5] Garcia-Vazquez FA, Gadea J, Matas C, Holt WV (2016). Importance of sperm morphology during sperm transport and fertilization in mammals. Asian J Androl.

[6] Williams HL, Mansell S, Alasmari W, Brown SG, Wilson SM, Sutton KA, Miller MR, Lishko PV, Barratt CL, Publicover SJ (2015). Specific loss of CatSper function is sufficient to compromise fertilizing capacity of human spermatozoa. Hum Reprod.

[7] Rodríguez-Martínez H (2013). Semen evaluation techniques and their relationship with fertility. Anim Reprod.

[8] Darr CR, Varner DD, Teague S, Cortopassi GA, Datta S, Meyers SA (2016). Lactate and pyruvate are major sources of energy for stallion sperm with dose effects on mitochondrial function, motility, and ROS production. Biol Reprod.

[9] Fair S, Lonergan P (2018). Review: understanding the causes of variation in reproductive wastage among bulls. Animal.

[10] Morrell JM, Nongbua T, Valeanu S, Lima Verde I, Lundstedt-Enkel K, Edman A, Johannisson A (2017). Sperm quality variables as indicators of bull fertility may be breed dependent. Anim Reprod Sci.

[11] Kasvandik S, Sillaste G, Velthut-Meikas A, Mikelsaar AV, Hallap T, Padrik P, Tenson T, Jaakma U, Koks S, Salumets A (2015). Bovine sperm plasma membrane proteomics through biotinylation and subcellular enrichment. Proteomics.

[12] Wood PL, Scoggin K, Ball BA, Troedsson MH, Squires EL (2016). Lipidomics of equine sperm and seminal plasma: identification of amphiphilic (O-acyl)-omega-hydroxy-fatty acids. Theriogenology.

[13] Camargo M, Intasqui P, Bertolla RP (2018). Understanding the seminal plasma proteome and its role in male fertility. Basic Clin Androl.

[14] Oliveira BM, Arruda RP, Thome HE, Maturana Filho M, Oliveira G, Guimaraes C, Nichi M, Silva LA, Celeghini EC (2014). Fertility and uterine hemodynamic in cows after artificial insemination with semen assessed by fluorescent probes. Theriogenology.

[15] Kwon WS, Rahman MS, Ryu DY, Park YJ, Pang MG (2015). Increased male fertility using fertility-related biomarkers. Sci Rep.

[16] Erikson DW, Way AL, Chapman DA, Killian GJ (2007). Detection of osteopontin on Holstein bull spermatozoa, in cauda epididymal fluid and testis homogenates, and its potential role in bovine fertilization. Reproduction.

[17] Killian GJ, Chapman DA, Rogowski LA (1993). Fertility-associated proteins in Holstein bull seminal plasma. Biol Reprod.

[18] Moura AA, Chapman DA, Killian GJ (2007). Proteins of the accessory sex glands associated with the oocyte-penetrating capacity of cauda epididymal sperm from Holstein bulls of documented fertility. Mol Reprod Dev.

[19] Moura AA, Chapman DA, Koc H, Killian GJ (2006). Proteins of the cauda epididymal fluid associated with fertility of mature dairy bulls. J Androl.

[20] Fagerlind M, Stalhammar H, Olsson B, Klinga-Levan K (2015). Expression of miRNAs in bull spermatozoa correlates with fertility rates. Reprod Domest Anim.

[21] Govindaraju A, Uzun A, Robertson L, Atli MO, Kaya A, Topper E, Crate EA, Padbury J, Perkins A, Memili E (2012). Dynamics of microRNAs in bull spermatozoa. Reprod Biol Endocrinol.

[22] Gromski PS, Muhamadali H, Ellis DI, Xu Y, Correa E, Turner ML, Goodacre R (2015). A tutorial review: metabolomics and partial least squares-discriminant analysis--a marriage of convenience or a shotgun wedding. Anal Chim Acta.

[23] Dipresa S, De Toni L, Foresta C, Garolla A (2018). New markers for predicting fertility of the male gametes in the post genomic age. Protein Pept Lett.

[24] Fukusaki E (2014). Application of Metabolomics for High Resolution Phenotype Analysis. Mass Spectrom (Tokyo).

[25] Guijas C, Montenegro-Burke JR, Warth B, Spilker ME, Siuzdak G (2018). Metabolomics activity screening for identifying metabolites that modulate phenotype. Nat Biotechnol.

[26] Odet F, Gabel S, London RE, Goldberg E, Eddy EM (2013). Glycolysis and mitochondrial respiration in mouse LDHC-null sperm. Biol Reprod.

[27] Tang B., Shang X., Qi H., Li J., Ma B., An G., Zhang Q. (2017). Metabonomic analysis of fatty acids in seminal plasma between healthy and asthenozoospermic men based on gas chromatography mass spectrometry. Andrologia.

[28] Qiao S, Wu W, Chen M, Tang Q, Xia Y, Jia W, Wang X (2017). Seminal plasma metabolomics approach for the diagnosis of unexplained male infertility. PLoS One.

[29] Kumar A, Kroetsch T, Blondin P, Anzar M (2015). Fertility-associated metabolites in bull seminal plasma and blood serum: 1H nuclear magnetic resonance analysis. Mol Reprod Dev.

[30] Velho ALC, Menezes E, Dinh T, Kaya A, Topper E, Moura AA, Memili E (2018). Metabolomic markers of fertility in bull seminal plasma. PLoS One.

[31] Paiva C, Amaral A, Rodriguez M, Canyellas N, Correig X, Ballesca JL, Ramalho-Santos J, Oliva R (2015). Identification of endogenous metabolites in human sperm cells using proton nuclear magnetic resonance ((1) H-NMR) spectroscopy and gas chromatography-mass spectrometry (GC-MS). Andrology.

[32] Zhao K, Zhang J, Xu Z, Xu Y, Xu A, Chen W, Miao C, Liu S, Wang Z, Jia R (2018). Metabolomic profiling of human spermatozoa in idiopathic Asthenozoospermia patients using gas chromatography-mass spectrometry. Biomed Res Int.

[33] Reynolds S, Calvert SJ, Paley MN, Pacey AA (2017). 1H magnetic resonance spectroscopy of live human sperm. Mol Hum Reprod.

[34] Holden SA, Fernandez-Fuertes B, Murphy C, Whelan H, O’Gorman A, Brennan L, Butler ST, Lonergan P, Fair S (2017). Relationship between in vitro sperm functional assessments, seminal plasma composition, and field fertility after AI with either non-sorted or sex-sorted bull semen. Theriogenology.

[35] Marin S, Chiang K, Bassilian S, Lee WN, Boros LG, Fernandez-Novell JM, Centelles JJ, Medrano A, Rodriguez-Gil JE, Cascante M (2003). Metabolic strategy of boar spermatozoa revealed by a metabolomic characterization. FEBS Lett.

[36] Patel AB, Srivastava S, Phadke RS, Govil G (1998). Arginine activates glycolysis of goat epididymal spermatozoa: an NMR study. Biophys J.

[37] Engel KM, Baumann S, Rolle-Kampczyk U, Schiller J, von Bergen M, Grunewald S (2019). Metabolomic profiling reveals correlations between spermiogram parameters and the metabolites present in human spermatozoa and seminal plasma. PLoS One.

[38] Sauer SW, Okun JG, Hoffmann GF, Koelker S, Morath MA (2008). Impact of short- and medium-chain organic acids, acylcarnitines, and acyl-CoAs on mitochondrial energy metabolism. Biochim Biophys Acta.

[39] Reynolds S, Ismail NFB, Calvert SJ, Pacey AA, Paley MNJ (2017). Evidence for rapid oxidative phosphorylation and lactate fermentation in motile human sperm by hyperpolarized (13)C magnetic resonance spectroscopy. Sci Rep.

[40] Iaffaldano N, Di Iorio M, Mannina L, Paventi G, Rosato MP, Cerolini S, Sobolev AP (2018). Age-dependent changes in metabolic profile of Turkey spermatozoa as assessed by NMR analysis. PLoS One.

[41] Gilany Kambiz, Mani-Varnosfaderani Ahmad, Minai-Tehrani Arash, Mirzajani Fateme, Ghassempour Alireza, Sadeghi Mohammed Reza, Amini Mehdi, Rezadoost Hassan (2017). Untargeted metabolomic profiling of seminal plasma in nonobstructive azoospermia men: A noninvasive detection of spermatogenesis. Biomedical Chromatography.

[42] Chen X, Hu C, Dai J, Chen L (2015). Metabolomics analysis of seminal plasma in infertile males with kidney-yang deficiency: a preliminary study. Evid Based Complement Alternat Med.

[43] Amaral A, Castillo J, Estanyol JM, Ballesca JL, Ramalho-Santos J, Oliva R (2013). Human sperm tail proteome suggests new endogenous metabolic pathways. Mol Cell Proteomics.

[44] Ferramosca A, Moscatelli N, Di Giacomo M, Zara V (2017). Dietary fatty acids influence sperm quality and function. Andrology.

[45] Visconti PE (2012). Sperm bioenergetics in a nutshell. Biol Reprod.

[46] Paventi G, Lessard C, Bailey JL, Passarella S (2015). In boar sperm capacitation L-lactate and succinate, but not pyruvate and citrate, contribute to the mitochondrial membrane potential increase as monitored via safranine O fluorescence. Biochem Biophys Res Commun.

[47] Piomboni P, Focarelli R, Stendardi A, Ferramosca A, Zara V (2012). The role of mitochondria in energy production for human sperm motility. Int J Androl.

[48] Garcia BM, Fernandez LG, Ferrusola CO, Salazar-Sandoval C, Rodriguez AM, Martinez HR, Tapia JA, Morcuende D, Pena FJ (2011). Membrane lipids of the stallion spermatozoon in relation to sperm quality and susceptibility to lipid peroxidation. Reprod Domest Anim.

[49] Waterhouse KE, Hofmo PO, Tverdal A, Miller RR (2006). Within and between breed differences in freezing tolerance and plasma membrane fatty acid composition of boar sperm. Reproduction.

[50] Alizadeh A, Esmaeili V, Shahverdi A, Rashidi L (2014). Dietary fish oil can change sperm parameters and fatty acid profiles of ram sperm during oil consumption period and after removal of oil source. Cell J.

[51] Khosrowbeygi A, Zarghami N (2007). Fatty acid composition of human spermatozoa and seminal plasma levels of oxidative stress biomarkers in subfertile males. Prostaglandins Leukot Essent Fatty Acids.

[52] Zerbinati C, Caponecchia L, Rago R, Leoncini E, Bottaccioli AG, Ciacciarelli M, Pacelli A, Salacone P, Sebastianelli A, Pastore A (2016). Fatty acids profiling reveals potential candidate markers of semen quality. Andrology.

[53] Martinez-Soto JC, Landeras J, Gadea J (2013). Spermatozoa and seminal plasma fatty acids as predictors of cryopreservation success. Andrology.

[54] Kiernan M, Fahey AG, Fair S (2013). The effect of the in vitro supplementation of exogenous long-chain fatty acids on bovine sperm cell function. Reprod Fertil Dev.

[55] Hossain MS, Afrose S, Sawada T, Hamano KI, Tsujii H (2010). Metabolism of exogenous fatty acids, fatty acid-mediated cholesterol efflux, PKA and PKC pathways in boar sperm acrosome reaction. Reprod Med Biol.

[56] Lundin M, Baltscheffsky H, Ronne H (1991). Yeast PPA2 gene encodes a mitochondrial inorganic pyrophosphatase that is essential for mitochondrial function. J Biol Chem.

[57] Yi YJ, Sutovsky M, Kennedy C, Sutovsky P (2012). Identification of the inorganic pyrophosphate metabolizing, ATP substituting pathway in mammalian spermatozoa. PLoS One.

[58] Amelar RD, Dubin L, Schoenfeld C (1980). Sperm motility. Fertil Steril.

[59] Fakih H, MacLusky N, DeCherney A, Wallimann T, Huszar G (1986). Enhancement of human sperm motility and velocity in vitro: effects of calcium and creatine phosphate. Fertil Steril.

[60] Schaefer WH (2006). Reaction of primary and secondary amines to form carbamic acid glucuronides. Curr Drug Metab.

[61] Meigh L, Greenhalgh SA, Rodgers TL, Cann MJ, Roper DI, Dale N (2013). CO(2) directly modulates connexin 26 by formation of carbamate bridges between subunits. Elife.

[62] Meigh L (2015). CO2 carbamylation of proteins as a mechanism in physiology. Biochem Soc Trans.

[63] Lorimer GH (1983). Carbon dioxide and carbamate formation: the makings of a biochemical control system. Trends Biochem Sci.

[64] Nishigaki T, Jose O, Gonzalez-Cota AL, Romero F, Trevino CL, Darszon A (2014). Intracellular pH in sperm physiology. Biochem Biophys Res Commun.

[65] Persson H, Pelto-Huikko M, Metsis M, Soder O, Brene S, Skog S, Hokfelt T, Ritzen EM (1990). Expression of the neurotransmitter-synthesizing enzyme glutamic acid decarboxylase in male germ cells. Mol Cell Biol.

[66] Ritta MN, Calamera JC, Bas DE (1998). Occurrence of GABA and GABA receptors in human spermatozoa. Mol Hum Reprod.

[67] Ritta MN, Bas DE, Tartaglione CM (2004). In vitro effect of gamma-aminobutyric acid on bovine spermatozoa capacitation. Mol Reprod Dev.

[68] Puente MA, Tartaglione CM, Ritta MN (2011). Bull sperm acrosome reaction induced by gamma-aminobutyric acid (GABA) is mediated by GABAergic receptors type a. Anim Reprod Sci.

[69] Jin JY, Chen WY, Zhou CX, Chen ZH, Yu-Ying Y, Ni Y, Chan HC, Shi QX (2009). Activation of GABAA receptor/cl- channel and capacitation in rat spermatozoa: HCO3- and cl- are essential. Syst Biol Reprod Med.

[70] de las Heras MA, Valcarcel A, Perez LJ (1997). In vitro capacitating effect of gamma-aminobutyric acid in ram spermatozoa. Biol Reprod.

[71] Calogero AE, Hall J, Fishel S, Green S, Hunter A, D'Agata R (1996). Effects of gamma-aminobutyric acid on human sperm motility and hyperactivation. Mol Hum Reprod.

[72] Kumar M, Dillon GH (2016). Assessment of direct gating and allosteric modulatory effects of meprobamate in recombinant GABA(a) receptors. Eur J Pharmacol.

[73] Pfennig T, Herrmann B, Bauer T, Schomig E, Grundemann D (2013). Benzoic acid and specific 2-oxo acids activate hepatic efflux of glutamate at OAT2. Biochim Biophys Acta.

[74] Langendorf CG, Tuck KL, Key TL, Fenalti G, Pike RN, Rosado CJ, Wong AS, Buckle AM, Law RH, Whisstock JC (2013). Structural characterization of the mechanism through which human glutamic acid decarboxylase auto-activates. Biosci Rep.

[75] Ebrahimi F, Ibrahim B, Teh CH, Murugaiyah V, Chan KL (2016). Urinary NMR-based metabolomic analysis of rats possessing variable sperm count following orally administered Eurycoma longifolia extracts of different quassinoid levels. J Ethnopharmacol.

[76] Odet F, Gabel SA, Williams J, London RE, Goldberg E, Eddy EM (2011). Lactate dehydrogenase C and energy metabolism in mouse sperm. Biol Reprod.

[77] du Plessis SS, Agarwal A, Mohanty G, van der Linde M (2015). Oxidative phosphorylation versus glycolysis: what fuel do spermatozoa use?. Asian J Androl.

[78] O'Flaherty CM, Beorlegui NB, Beconi MT (2002). Lactate dehydrogenase-C4 is involved in heparin- and NADH-dependent bovine sperm capacitation. Andrologia.

[79] Tang H, Duan C, Bleher R, Goldberg E (2013). Human lactate dehydrogenase a (LDHA) rescues mouse Ldhc-null sperm function. Biol Reprod.

[80] Odet F, Duan C, Willis WD, Goulding EH, Kung A, Eddy EM, Goldberg E (2008). Expression of the gene for mouse lactate dehydrogenase C (Ldhc) is required for male fertility. Biol Reprod.

[81] Zhu Z, Li R, Ma G, Bai W, Fan X, Lv Y, Luo J, Zeng W (2018). 5′-AMP-activated protein kinase regulates goat sperm functions via energy metabolism in vitro. Cell Physiol Biochem.

[82] Miro J, Lobo V, Quintero-Moreno A, Medrano A, Pena A, Rigau T (2005). Sperm motility patterns and metabolism in Catalonian donkey semen. Theriogenology.

[83] Andersen JM, Ronning PO, Herning H, Bekken SD, Haugen TB, Witczak O (2016). Fatty acid composition of spermatozoa is associated with BMI and with semen quality. Andrology.

[84] Esmaeili V, Shahverdi AH, Moghadasian MH, Alizadeh AR (2015). Dietary fatty acids affect semen quality: a review. Andrology.

[85] Tavilani H, Doosti M, Abdi K, Vaisiraygani A, Joshaghani HR (2006). Decreased polyunsaturated and increased saturated fatty acid concentration in spermatozoa from asthenozoospermic males as compared with normozoospermic males. Andrologia.

[86] Rana AP, Majumder GC, Misra S, Ghosh A (1991). Lipid changes of goat sperm plasma membrane during epididymal maturation. Biochim Biophys Acta.

[87] Hu SG, Liang AJ, Yao GX, Li XQ, Zou M, Liu JW, Sun Y (2018). The dynamic metabolomic changes throughout mouse epididymal lumen fluid potentially contribute to sperm maturation. Andrology.

[88] Flegel C, Vogel F, Hofreuter A, Schreiner BS, Osthold S, Veitinger S, Becker C, Brockmeyer NH, Muschol M, Wennemuth G (2015). Characterization of the olfactory receptors expressed in human spermatozoa. Front Mol Biosci.

[89] Adipietro KA, Mainland JD, Matsunami H (2012). Functional evolution of mammalian odorant receptors. PLoS Genet.

[90] Massberg D, Jovancevic N, Offermann A, Simon A, Baniahmad A, Perner S, Pungsrinont T, Luko K, Philippou S, Ubrig B (2016). The activation of OR51E1 causes growth suppression of human prostate cancer cells. Oncotarget.

[91] Flegel C, Vogel F, Hofreuter A, Wojcik S, Schoeder C, Kiec-Kononowicz K, Brockmeyer NH, Muller CE, Becker C, Altmuller J (2016). Characterization of non-olfactory GPCRs in human sperm with a focus on GPR18. Sci Rep.

[92] Davis JT, Bridges RB, Coniglio JG (1966). Changes in lipid composition of the maturing rat testis. Biochem J.

[93] Goodson SG, Qiu Y, Sutton KA, Xie G, Jia W, O'Brien DA (2012). Metabolic substrates exhibit differential effects on functional parameters of mouse sperm capacitation. Biol Reprod.

[94] Litvinov D, Selvarajan K, Garelnabi M, Brophy L, Parthasarathy S (2010). Anti-atherosclerotic actions of azelaic acid, an end product of linoleic acid peroxidation, in mice. Atherosclerosis.

[95] Jones DA (2009). Rosacea, reactive oxygen species, and azelaic acid. J Clin Aesthet Dermatol.

[96] Schallreuter KU, Wood JW (1990). A possible mechanism of action for azelaic acid in the human epidermis. Arch Dermatol Res.

[97] Passi S, Picardo M, Mingrone G, Breathnach AS, Nazzaro-Porro M (1989). Azelaic acid--biochemistry and metabolism. Acta Derm Venereol Suppl (Stockh).

[98] Breathnach AS (1999). Azelaic acid: potential as a general antitumoural agent. Med Hypotheses.

[99] Fitton A, Goa KL (1991). Azelaic acid. A review of its pharmacological properties and therapeutic efficacy in acne and hyperpigmentary skin disorders. Drugs.

[100] Muthulakshmi S, Saravanan R (2013). Efficacy of azelaic acid on hepatic key enzymes of carbohydrate metabolism in high fat diet induced type 2 diabetic mice. Biochimie.

[101] Hou Y, Yin Y, Wu G (2015). Dietary essentiality of “nutritionally non-essential amino acids” for animals and humans. Exp Biol Med (Maywood).

[102] Yuan YY, He CN, Shi QX (1998). GABA initiates the acrosome reaction and fertilizing ability in human sperm. Sheng Li Xue Bao.

[103] Tiedje KE, Stevens K, Barnes S, Weaver DF (2010). Beta-alanine as a small molecule neurotransmitter. Neurochem Int.

[104] Pelliccione F, Micillo A, Cordeschi G, D'Angeli A, Necozione S, Gandini L, Lenzi A, Francavilla F, Francavilla S (2011). Altered ultrastructure of mitochondrial membranes is strongly associated with unexplained asthenozoospermia. Fertil Steril.

[105] Peddinti D, Nanduri B, Kaya A, Feugang JM, Burgess SC, Memili E (2008). Comprehensive proteomic analysis of bovine spermatozoa of varying fertility rates and identification of biomarkers associated with fertility. BMC Syst Biol.

[106] Zwald NR, Weigel KA, Chang YM, Welper RD, Clay JS (2004). Genetic selection for health traits using producer-recorded data. II. Genetic correlations, disease probabilities, and relationships with existing traits. J Dairy Sci.

[107] Zwald NR, Weigel KA, Chang YM, Welper RD, Clay JS (2004). Genetic selection for health traits using producer-recorded data. I. Incidence rates, heritability estimates, and sire breeding values. J Dairy Sci.

[108] Chang YM, Gianola D, Heringstad B, Klemetsdal G (2014). Effects of trait definition on genetic parameter estimates and sire evaluation for clinical mastitis with threshold models. Anim Sci.

[109] Wishart DS, Tzur D, Knox C, Eisner R, Guo AC, Young N, Cheng D, Jewell K, Arndt D, Sawhney S (2007). HMDB: the human metabolome database. Nucleic Acids Res.

[110] Wishart DS, Jewison T, Guo AC, Wilson M, Knox C, Liu Y, Djoumbou Y, Mandal R, Aziat F, Dong E (2013). HMDB 3.0--the human metabolome database in 2013. Nucleic Acids Res.

[111] Chong J, Soufan O, Li C, Caraus I, Li S, Bourque G, Wishart DS, Xia J (2018). MetaboAnalyst 4.0: towards more transparent and integrative metabolomics analysis. Nucleic Acids Res.

[112] Xia J, Broadhurst DI, Wilson M, Wishart DS (2013). Translational biomarker discovery in clinical metabolomics: an introductory tutorial. Metabolomics.

[113] Xia J, Wishart DS (2011). Metabolomic data processing, analysis, and interpretation using MetaboAnalyst. Curr Protoc Bioinformatics.

[114] Xia J, Wishart DS (2010). MetPA: a web-based metabolomics tool for pathway analysis and visualization. Bioinformatics.

